# Wider psychische Belastungen – resilientes Arbeiten in der Chirurgie

**DOI:** 10.1007/s00104-023-01977-9

**Published:** 2023-11-21

**Authors:** Beatrice Thielmann, Frank Meyer, Irina Böckelmann

**Affiliations:** 1 Bereich Arbeitsmedizin, Medizinische Fakultät, Otto-von-Guericke-Universität Magdeburg, Magdeburg, Deutschland; 2 Universitätsklinik für Allgemein-, Viszeral-, Gefäß- und Transplantationschirurgie, Otto-von-Guericke-Universität Magdeburg mit Universitätsklinikum Magdeburg A. ö. R., Magdeburg, Deutschland

**Keywords:** Kohärenzgefühl, Selbstwirksamkeitserwartung, Resilienzfaktor, Psychische Beanspruchung, Soziale Unterstützung, Sense of coherence, Self-efficacy expectation, Resilience (factor), Psychological stress, Social support

## Abstract

**Hintergrund:**

Die Chirurgie stellt eine anspruchsvolle medizinische Fachdisziplin dar.

**Ziel:**

Dieser Artikel befasst sich, basierend auf selektiven Referenzen der medizinisch-wissenschaftlichen Literatur und eigenen einschlägigen Erfahrungen aus dem klinischen Alltag, mit psychischen Belastungen in der Chirurgie und erklärt Resilienz als Schutzfaktor gegenüber psychischen Beanspruchungsfolgen. Dabei werden das Kohärenzgefühl, soziale Unterstützung und die Selbstwirksamkeitserwartung als Resilienzfaktoren näher erörtert.

**Methode:**

Narrative Übersicht.

**Ergebnisse (Eckpunkte):**

Die Chirurgie wird als herausforderndes medizinisches Teilgebiet mit hohem Ansehen eingestuft, jedoch mit diversen und vielfältigen physischen und psychischen Belastungen assoziiert. Belastungsfaktoren differieren individuell durch Anforderungen (können über- oder unterfordern/jedoch auch anregend, lernrelevant und sinnstiftend sein, damit positiv oder negativ belastend wirken) und Ressourcen (potenziell förderliche Arbeitsbedingungen, Erfahrungen oder Verhaltensweisen – bspw. soziale Unterstützung, Handlungsspielräume etc.). Fluktuationen innerhalb der chirurgischen Fächer und eine hohe Abbruchrate während der Facharztausbildung sind hinreichend bekannt – ursächlich sind u. a. hohe psychische Belastungen. Bei langanhaltender und zeitgleich unzureichender Kompensation von Arbeitsbelastungen infolge fehlender oder ungenügender Ressourcen können diese mit psychischen Erkrankungen verbunden sein. Nichtdestotrotz verbringen viele Ärzte zeitlebens ihre Arbeit im klinischen oder niedergelassenen chirurgischen Setting und bleiben trotzdem gesund – eine stark ausgeprägte Resilienz gegenüber psychischen Erkrankungen kann grundlegend dafür sein. Resilienz kann dabei als persönliche Eigenschaft vorhanden sein oder durch einen Prozess erlernt werden bzw. durch positive oder negative Einflüsse angepasst sein und damit die persönliche Eigenschaft stärken. Insgesamt sind Daten über die Resilienz der Chirurgen bzw. über Interventionsstudien in der Resilienzforschung im Setting Chirurgie limitiert und bieten eine weitere Forschungslücke. Resilienztraining (gerichtet auf Kohärenzsinn, soziale Unterstützung, Stärkung des Wissens über Bewältigungsmöglichkeiten, positive Emotionen, Optimismus, Hoffnung, Selbstwirksamkeitserwartung, Kontrollüberzeugungen oder Robustheit) – auch klar angezeigt im „robusten“ medizinisch-operativen Fach Chirurgie – ist immer individuell und sollte nicht pauschalisiert werden. Wenn der Chirurg aufgrund der Belastungssituation nicht ausreichend Ressourcen abrufen kann, ist ein Stressmanagement mit seinen Methoden hilfreich, um den psychisch belastenden Stress zu verringern und um die Leistungsfähigkeit und Gesundheit dieser Person erhalten zu können.

**Schlussfolgerung:**

Die Konsolidierung der Resilienz ist ein beachtenswerter Aspekt der Mitarbeiterführung. Im interkollegialen Umgang muss sich Resilienz auf arbeitsplatzbezogene Ansätze stützen zur Stärkung der Bewältigungsmechanismen gegenüber Arbeitsbelastungen. Arbeitsplatzbedingte Belastungen sollten auch – durchaus auch als elementare Leitungsaufgabe – unternehmensintern wahrgenommen, angesprochen und entgegengewirkt werden.

## Abstract (en)

**Background:**

Surgery represents a challenging medical discipline.

**Aim:**

This article focuses on psychological stress in surgery and explains resilience as a protective factor against the consequences of psychological stress, based on selected literature references and own relevant clinical experiences. In this context, the sense of coherence, social support and self-efficacy expectation are discussed in more detail as resilience factors.

**Results (corner points):**

Surgery is classified as a challenging medical subspecialty with a high reputation but associated with diverse and varied physical and psychological stress factors. Stress factors differ individually in terms of requirements (can be overdemanding or underdemanding but also stimulating, relevant to learning and meaningful, thus positively or negatively stressful) and resources (potentially beneficial working conditions, experience, or behavior, e.g., social support, scope for action). Fluctuations within surgical specialties and a high dropout rate during residency training are well known and the causes include high psychological stress. In the case of persistent and at the same time insufficient compensation of work stress caused by a lack of or insufficient resources, these can be associated with mental illnesses. Nonetheless, many physicians spend their entire lives working in hospital or private surgical settings and remain healthy, a strong sense of resilience to mental illness may be fundamental to this. Resilience can be present as a personal characteristic or it can be learnt through a process or adapted through positive or negative influences, thus strengthening the personal characteristics. Overall, data on surgeon resilience or interventional studies in resilience research in the surgical setting are limited and provide another research gap. Resilience training (directed at a sense of coherence, social support, strengthening knowledge of coping skills, positive emotions, optimism, hope, self-efficacy expectations, control beliefs or robustness), also clearly indicated in the “robust” medical specialty of surgery, is always individual and should not be generalized. If the surgeon cannot retrieve sufficient resources due to the stressful situation, stress management with its methods is helpful to reduce the psychological stress and to be able to maintain the performance and health of this person.

**Conclusion:**

The consolidation of resilience as a notable aspect of employee management. In collegial interactions, resilience must be based on workplace-based approaches to strengthen coping mechanisms in the face of work stress. Workplace-related stress should also be perceived, addressed and counteracted within the organization, certainly also as an elementary management task.

## Hintergrund

Die Chirurgie ist eine hoch anspruchsvolle und unter Medizinstudierenden und Absolventen der Humanmedizin angesehene medizinische Fachdisziplin. Nichtdestotrotz bestehen vielseitige physische und psychische Herausforderungen, die zum vorzeitigen Ausscheiden von Assistenzärzten^1^ während ihrer Facharztausbildung (aber auch zu diversen Zeitpunkten einer teils durchaus erfüllten Berufslaufbahn) führen, da eine Unvereinbarkeit von körperlicher, psychischer und auch spiritueller Lebensvision und somit ein unzureichendes Wohlbefinden besteht [1]. Fluktuationen in der Chirurgie liegen dabei im Mittel um 18 %, wobei diese bei Frauen mit 25 % höher als bei Männern mit 15 % [2] sind. Dabei erfolgt am häufigsten ein Wechsel in ein anderes allgemeinchirurgisches Programm oder in die Anästhesie nach ca. einem Weiterbildungsjahr [2]. Ein Hauptgrund für das Ausscheiden ist einerseits ein unvorhersehbarer Lebensstil oder die (entdeckte oder entwickelte bzw. klinisch kompetenter eruierte) Vorliebe zu einem anderen chirurgischen Fach oder einer anderen medizinischen Teildisziplin [2].

### Ziel.

Dieser Artikel befasst sich, basierend auf selektiven Referenzen der medizinisch-wissenschaftlichen Literatur und eigenen einschlägigen Erfahrungen aus dem klinischen Alltag, mit psychischen Belastungen in der Chirurgie und erklärt Resilienz als Schutzfaktor gegenüber psychischen Beanspruchungsfolgen. Dabei werden das Kohärenzgefühl, soziale Unterstützung und die Selbstwirksamkeitserwartung als Resilienzfaktoren näher besprochen.

## Methode

Es wurde für ein narratives Review durchgeführt und in diesem Zusammenhang in elektronischen Datenbanken MEDLINE/PubMed und Psyndex nationale und internationale Publikationen der letzten 20 Jahre mit den Suchwörtern „Resilienz“ und „Chirurgie“ gesichtet. Fokussiert wurde auf Resilienz der Chirurgen, Interventionsstudien in der Resilienzforschung im Setting Chirurgie und allgemeines Resilienztraining.

Da es sich um ein narratives Review handelt, wurden die Ergebnisse dieser Literatursuche ohne eine klare wissenschaftliche Methodik, wie es für das systematische Review oder Metaanalysen üblich ist, angefertigt. Die Sichtung der Literatur erfolgte nur in deutsch- oder englischsprachigen Publikationen.

## Ergebnisse (inhaltliche Eckpunkte)

### Belastung und Beanspruchung kurz rekapituliert

**Figure 1:**
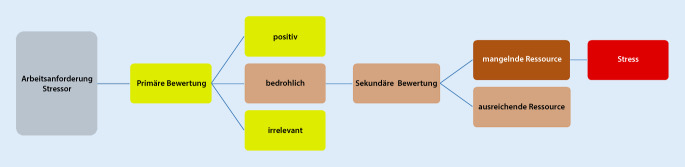

Psychische Belastung wird in der DIN EN ISO 10075‑1 als „… die Gesamtheit aller erfassbaren Einflüsse, die von außen auf den Menschen zukommen und psychisch auf ihn einwirken“ definiert. Psychische Beanspruchung weist auf die Auswirkungen des Individuums hin und wird nach DIN EN ISO 10075‑1 definiert als „… die unmittelbare (nicht die langfristige) Auswirkung der psychischen Belastung im Individuum in Abhängigkeit von seinen jeweiligen überdauernden und augenblicklichen Voraussetzungen, einschließlich der individuellen Bewältigungsstrategien“. Eine psychische Belastungssituation kann auf unterschiedliche Faktoren (z. B. Schichtarbeit, fehlende soziale Unterstützung, hohe Arbeitsintensität) zurückgeführt werden (Belastungsfaktoren). Sie werden in der Arbeitsmedizin/-wissenschaft wertneutral, also weder positiv noch negativ verstanden [3]. Grundlegend dafür ist, dass diese Belastungsfaktoren durch Anforderungen und Ressourcen individuell differieren. Die zu bewältigenden „Anforderungen können über- oder unterfordern. Sie können jedoch auch anregend, lernrelevant und sinnstiftend sein“ [4]. Potenziell förderliche Arbeitsbedingungen, Erfahrungen oder Verhaltensweisen werden als Ressource verstanden und sind bspw. soziale Unterstützung, Handlungsspielräume etc. [3]. Es kommt somit zu einer Bewertung der Arbeitsanforderung, die positiv oder negativ belastend wirken kann. Dies kann man gut mit dem transaktionellen Stressmodell nach Lazarus und Launier erklären [5]. Dieses Modell ist in der Abb. 1 dargestellt. Durch die primäre Bewertung der Arbeitsanforderung bzw. des Stressors (positiv, bedrohlich oder irrelevant) sowie die sekundäre Bewertung in Bezug auf die Ressource (mangelnde oder ausreichende) wird dieser als Stress oder nicht als Stress wahrgenommen.

**Figure 2:**
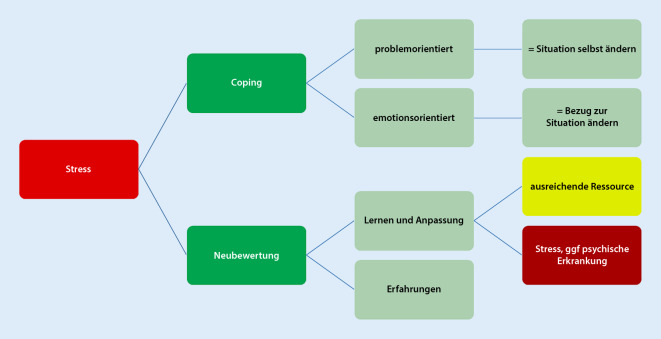

Wie in Abb. 2 dargestellt, kann durch Stressbewältigung eine Anpassung an den Stress erfolgen. Ergänzend anzumerken ist, dass eine Vielzahl von Stressmodellen und Konzepten entwickelt wurden, die einen Zusammenhang zwischen den Belastungen/Stressoren und der Gesundheit erklären und die einzeln auch im chirurgischen Setting angewandt werden können.

### Typische psychische Belastungen im Arztberuf und in der Chirurgie – eine Auswahl an Studienergebnissen

Psychische Belastungen in der chirurgischen Arbeitswelt spielen eine wichtige Schlüsselrolle für Gesundheit und Leistungsfähigkeit des pflegerischen und ärztlichen Personals. Als typische psychische arbeitsbedingte Belastungsfaktoren im Arztberuf werden häufig genannt: lange und oft ungeregelte Arbeitszeit, Überstunden, Bereitschafts- und Schichtdienste sowie dementsprechend zu wenig Freizeit, hohe Verantwortung, Zeitdruck, hoher Dokumentations- und Bürokratieaufwand, unzureichende Anerkennung seitens der Führung und unzureichendes Feedback für geleistete Arbeit, unzureichender Arbeitslohn, hoher Erwartungsdruck, Angst vor Behandlungs- und Kunstfehlern mit juristischen Folgen sowie Gratifikationskrisen [6] und auch lange studienbasierte und berufliche Entwicklungswege und Qualifikationskurrikula sowie anspruchsvolle bzw. teils extrem herausfordernde „Aufstiegs“chancen. Des Weiteren führen hohe Patientenerwartungen zur psychischen Beanspruchung [7].

Eine Studie über 643 deutsche Chirurgen (35 % weiblich) befasste sich mit Stressoren, Ressourcen und psychischem Befinden [8]. Dabei fanden sich interessanterweise keine relevanten Unterschiede zwischen den einzelnen chirurgischen Fachdisziplinen. Typische Stressoren waren

- arbeitsorganisatorische Probleme durch schlechte Arbeitsorganisation oder Arbeitsmittel,
- Arbeitsunterbrechungen,
- Widersprüche oder Unsicherheiten bei der Zielerreichung sowie ausbleibendes Feedback,
- Zeitdruck u. a. durch übermäßiges Arbeitsvolumen (und)
- hohe Konzentrationsanforderungen für längeren Zeitraum.

Als Ressourcen wurden die Arbeitskomplexität, a. e. durch Entscheidungsfindung, und der Handlungsspielraum erfasst. Als „Output“ wurde das psychische Befinden in Form von psychosomatischen Beschwerden, emotionaler Erschöpfung und Arbeitszufriedenheit ermittelt.

- Für Assistenz- und Fachärzte in Kliniken ergaben sich hohe Stressoren und wenig Handlungsspielraum. Das resultierte in einem schlechteren psychischen Befinden.
- Dagegen boten Chefärzte oder niedergelassene Ärzte eine hohe Arbeitszufriedenheit.

Zusammenfassend kam die Studie zur Aussage, das kleinere Organisationseinheiten günstigere Stressoren-Ressourcen-Kombinationen als große Krankenhäuser aufweisen [8]. Ein narratives Review über Assistenzärzte (äquivalent wie „Lernende im OP-Saal“) zeigte Ursachen für Stress wie Müdigkeit, Unterbrechungen, zwischenmenschliche Konflikte, Zeitdruck, einen komplexen Fall oder einen Hochrisikopatienten, chirurgische Fehler und das Temperament des Chirurgen auf [9].

Die stattgehabte SARS-CoV-2-Pandemie war dahingehend eine besondere Herausforderung und bot auch typische psychische Beanspruchungen wie die Angst vor einer Ansteckung und Erkrankung an COVID-19 [10]. Zu den klinischen Belastungsfaktoren gehörten die erhöhte klinische Belastung durch die Isolierung von Patienten und die hygienischen Maßnahmen, fehlendes Wissen und die begrenzte Berufserfahrung mit der Erkrankung als auch ein Rückgang der operativen Fälle [10]. Eine pandemiebedingte unfreiwillige Versetzung auf COVID-Stationen war ebenfalls ein psychischer Belastungsfaktor [10]. Des Weiteren war durch den Rückgang der Operationen während der Pandemie eine negative Auswirkung auf die chirurgische Ausbildung durch weniger praktische Erfahrung und Nichterfüllung der Fallanforderungen zu verzeichnen [10]. Die SARS-CoV-2-Pandemie hat aufgezeigt, wie sehr die Chirurgie VUCA(H) ist [11].

### Die Chirurgie ist VUCA(H)

VUCA(H) stellt ein Rahmenwerk für Veränderungsprozesse dar [12] und spielt somit im Gesundheitssektor eine enorme Rolle, da bspw. jeder Patient personenbezogen beurteilt wird. Es beschreibt Situationen, in denen Personen rasch und unkonventionell erkennen, beurteilen und handeln müssen. Grundsätzlich widerspricht es unserem Bedürfnis u. a. nach Sicherheit oder Klarheit, was in der Chirurgie z. B. in Standard Operating Procedure (SOP) beschrieben wird. VUCA(H) tritt bei jedem neuen Patientenkontakt auf, da dieser nicht pauschalisiert werden kann.

Das Akronym „VUCA(H)“ steht für „volatility“, „uncertainty“, „complexity“ und „ambiguity“. Da die Digitalisierung und Vernetzung eine treibende Kraft der VUCA-Welt ist, spricht man häufig auch noch von „hyper-connectivity“ [13]. Die Entstehung von VUCA ist dem US-Militär zuzusprechen [12, 14]. Dabei kam es aufgrund neuer Herausforderung durch neue Terrororganisationen zum Umdenken klassischer Militärführung. Ziel war es dabei, Entscheidungszyklen zu verkürzen, um Menschenleben zu retten [14].

**Figure 3:**
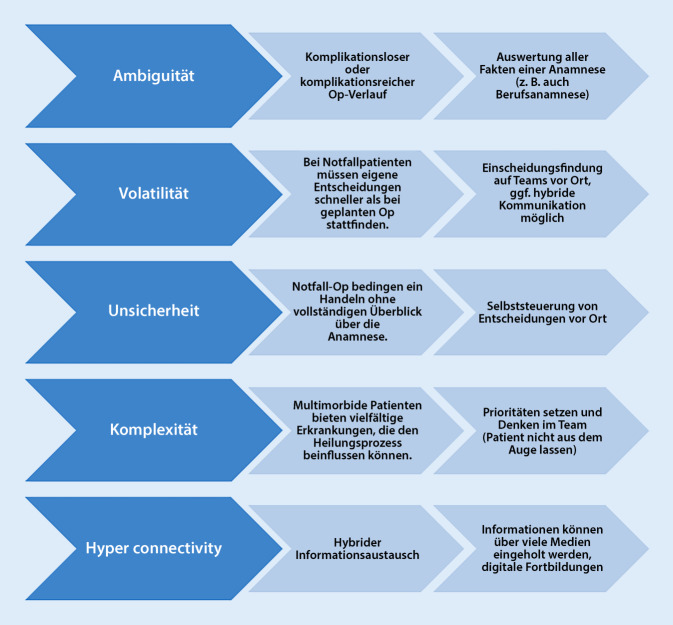

Eine Übertragung der VUCA(H)-Rahmenbedingungen ist auch auf die Chirurgie möglich und ist beispielhaft in der Abb. 3 dargestellt.

**Figure 4:**
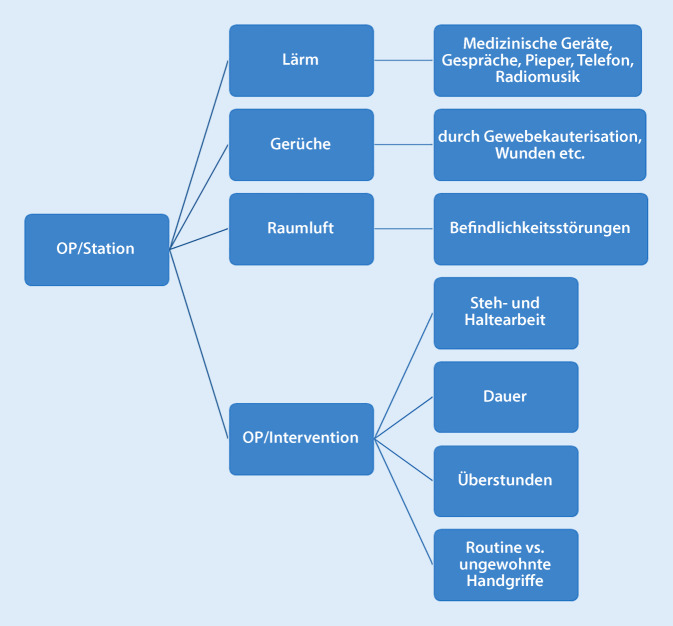

**Figure 5:**
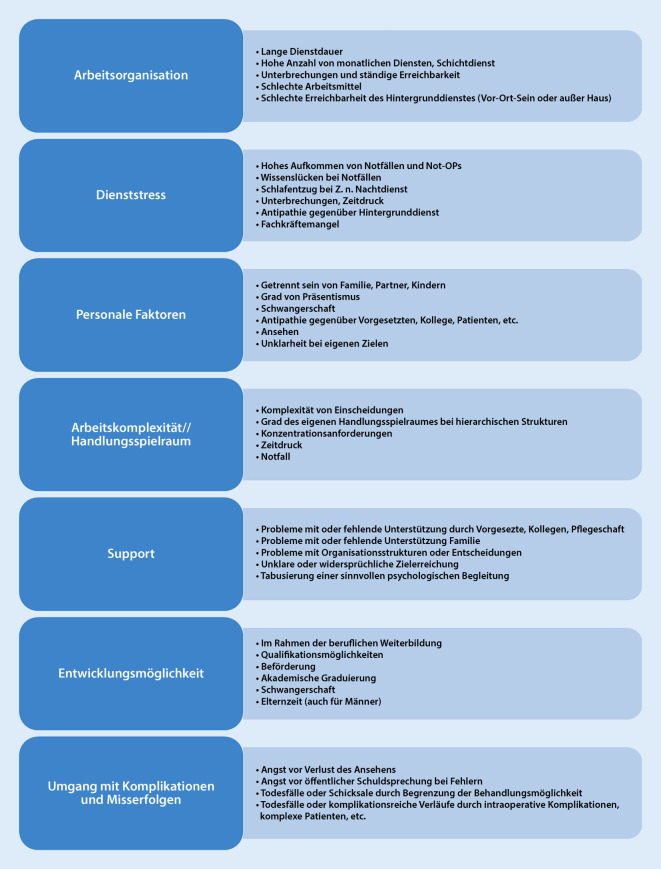

Prinzipiell lässt sich die in Abb. 3 dargestellte VUCA(H)-Welt auf viele Belastungen im chirurgischen Alltag übertragen. Die Autorengruppe stellt in Abb. 4 typische physische und in der Abb. 5 typische psychische Arbeitsbelastungen in der Chirurgie dar, die psychisch verbreitet werden und je nach Möglichkeiten der Stressbewältigung und resilientem Verhalten gut oder weniger gut kompensiert werden können. Darius et al. haben typische physische Belastungen in der Chirurgie dargestellt [16].

### Folgen psychischer Beanspruchung

**Figure 6:**
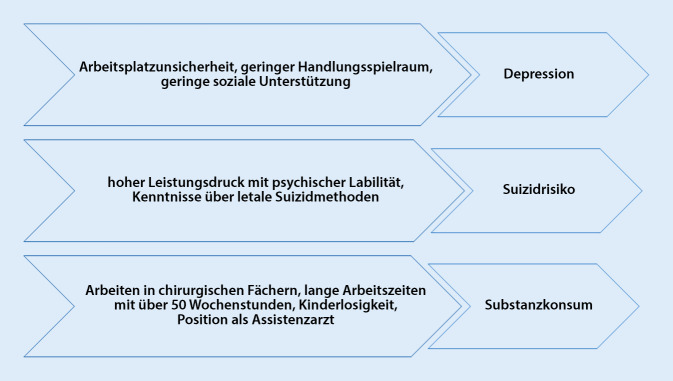

Psychische Belastung und als Folge dessen die psychische Beanspruchung bzw. die psychische Erkrankung werden häufig auch bei Ärzten schambesetzt und tabuisiert. Sie führen häufig zum Präsentismus, was wiederum weitere Gesundheitsgefahren birgt [17]. Eine unzureichende Kompensation langanhaltender Fehlbeanspruchung führt nicht selten zur Entwicklung psychischer Erkrankungen wie bspw. Depression, Suizidrisiko, Substanzkonsum oder Burnout [6]. Dabei konnten in Studien typische psychische Belastungen für psychische Erkrankungen in Arztberufen aufgezeigt werden [7, 18–23]. Diese sind in Abb. 6 aufgeführt.

In der Situation, in der der Chirurg aufgrund der Belastungssituation nicht ausreichend Ressourcen abrufen kann, ist ein Stressmanagement mit seinen Methoden hilfreich, um den psychisch belastenden Stress zu verringern und um die Leistungsfähigkeit und Gesundheit dieser Person erhalten zu können [3]. In diesem Zusammenhang ist besonders wichtig, resilient, d. h. mithilfe eigener Fähigkeiten, der Belastungssituation durch Rückgriff auf persönliche und sozial vermittelte Ressourcen entgegenzuwirken.

### Resilienz – kurz rekapituliert

„Resiliere“ bedeutet „zurückspringen“, „abprallen“. Zu den ersten Resilienzforschungen gehörte die Beobachtungsstudie von Werner und Smith, die 1955 in Hawaii begann. Viele Kinder waren trotz schlechter Lebensbedingungen während sie aufwuchsen im Verlauf über 40 Jahre Beobachtungszeit psychisch gesund und sozial integriert [24]. Die Forscher verwiesen dabei auf schützende Faktoren in der Familie, im Umfeld und bei den eigenen Persönlichkeitsmerkmalen. Diese Studie war u. a. Basis weiterer Forschungsfragen wie bspw., warum Menschen trotz Traumatisierungen gesund bleiben und diese Krise als Chance sehen können und warum andere Menschen dies nicht können [24]. Somit beschäftigt sich die Resilienzforschung mit der Frage, welche Faktoren relevant sind, um adäquat mit den Herausforderungen des Alltages und Berufes umzugehen und eine Balance zwischen Anforderungen und Ressourcen zu finden [24]. Resilienz kann dabei als persönliche Eigenschaft vorhanden sein oder durch einen Prozess erlernt bzw. durch die persönliche Eigenschaft gestärkt werden [25], wobei im wissenschaftlichen Konsens überwiegend der Prozess einer Resilienz betrachtet wird, der durch positive oder negative Einflüsse angepasst wird. Daher entwickelten sich unterschiedliche Definitionen von Resilienz [26–31]. Eine Auswahl an Definitionen ist Tab. 1 zu entnehmen. Sie stellt einen Verlauf über die Zeit dar und zeigt die Anpassungen der verschiedenen Definitionen. Allen Definitionen gemein ist, dass psychische Gesundheit trotz Widrigkeiten schnell wieder hergestellt wird oder aufrechterhalten bleibt. Resilienz ist laut der American Psychiatric Association (APA) ein Ergebnis der erfolgreichen Anpassung an schwierige oder herausfordernde Lebenserfahrungen, insbesondere durch mentale, emotionale und verhaltensmäßige Flexibilität und Anpassung an externe und interne Anforderungen [32]. Bis heute wird eine große Bandbreite an genetischen, psychologischen, sozialen und umweltbedingten Faktoren diskutiert, die als Resilienzfaktoren interagieren können [33].

**Table 1:** 

Autor	Wesentlicher Definitionsinhalt
Werner und Smith 1989	Resilienz wurde zuerst als eine stabile Persönlichkeitseigenschaft angesehen, auf die zusätzliche äußere Schutzfaktoren (z. B. soziale Unterstützung) einwirkten. Bedingt durch die Verlaufsbeobachtungen betrachteten die Autoren später die Resilienz auch als einen dynamischen Prozess [26]
Luthar et al. 2000	Die positive dynamische Anpassung an Umstände sind abhängig von der Schwere der Bedrohung und eine notwendige Leistung, sich positiv an die Herausforderung anzupassen [29]
Tugade und Fredrickson 2004	Resilienz als die emotionale Fähigkeit und flexible Anpassung an stressvolle Erfahrungen. Das Erleben positiver Gefühle/Erinnerungen und sozialer Interaktionen sowie Kreativität spielen eine große Rolle. Es gibt eine Schnittstelle zur positiven Psychologie [30]
Bengel und Lyssenko 2012	Resilienz ist multifaktoriell und kann durch Einzel- oder Gruppeninterventionen verbessert werden. Die Interventionsprogramme sind ebenfalls vielfältig, jedoch gibt es noch keinen empirisch abgesicherten theoretischen Rahmen [31]
Hartwig et al. 2016	Organisationale Resilienz bedeutet Funktionalität einer Organisation, sich aufrechtzuerhalten oder nach einer Störung schnellstmöglich wieder herzustellen [34]
Kalisch et al. 2017	Resilienz bedeutet Aufrechterhaltung oder schnelle Wiederherstellung der psychischen Gesundheit und wird als ein Prozess durch Anpassung an Stressoren gesehen [27]
Stainton et al. 2019	Schutzfaktoren und Ressourcen variieren innerhalb einer Person und sind somit abhängig von Zeit und Gegebenheiten. Resilienz ist damit ein dynamischer Prozess [28]
Thun-Hohenstein et al. 2020	Resilienz als ein Wechselspiel basaler humaner, adaptiver Systeme mit der Umwelt, um den Menschen zu befähigen, schwierige Lebenssituation zu bewältigen. Dabei müssen diese basalen Systeme geschützt und gepflegt werden, um sich robust zu entwickeln [35]

In der Arbeitsmedizin hat der Begriff *organisationale Resilienz* einen hohen Stellenwert. Dabei bestehen auf der Ebene der Arbeitsorganisation Strukturen und Prozesse, die gestaltbar und anpassungsfähig sind und dazu beitragen, dass eine Organisation gegenüber einer dynamischen Umwelt resilient ist. Die Bundesanstalt für Arbeitsschutz und Arbeitsmedizin definiert resilient als „die Funktionalität einer Organisation aufrechtzuerhalten oder nach einer Störung schnellstmöglich wieder herzustellen“ [34].

### Resilient(es) Arbeiten in der Chirurgie

Resilienz schützt vor Stress und Folgen wie Burnout. Resilienztraining erscheint nützlich, um Stress zu reduzieren und somit die psychische Gesundheit von Ärzten und Humanmedizinstudierenden zu verbessern [9]. Dabei gibt es verschiedene Ansätze, um Resilienz zu stärken [31]. Gestärkt bzw. trainiert werden können u. a. Kohärenzsinn, soziale Unterstützung, Stärkung des Wissens über Bewältigungsmöglichkeiten, positive Emotionen, Optimismus, Hoffnung, Selbstwirksamkeitserwartung, Kontrollüberzeugungen oder Robustheit [31].

Exemplarisch werden der Kohärenzsinn, die soziale Unterstützung und die Selbstwirksamkeitserwartung als Resilienzfaktoren näher erläutert.

- Der *Kohärenzsinn* kann nach Antonovsky als eine Überzeugung einer Person gesehen werden, das eigene Leben als sinnvoll, überschaubar und handhabbar wahrzunehmen. Ein hoher Kohärenzsinn nach Antonovsky erhöht die persönliche Stressresistenz, somit wird auch das persönliche Scheitern besser verarbeitet und beeinflusst, unangenehme Aspekte der Arbeit anzunehmen [36]. Der Kohärenzsinn enthält das Konzept des Kohärenzgefühls („sense of coherence“, SoC), welches drei Komponenten integriert: Verstehbarkeit, Handhabbarkeit und Sinnhaftigkeit (Abb. 5; [36]). Das Ausmaß der Wahrnehmung des eigenen Lebens (Kohärenzgefühl) ist ein entscheidender Parameter dafür, welche Positionierung diese Person auf dem Gesundheits-Krankheits-Kontinuum einnimmt. So bezeichnet die Handhabbarkeit, inwieweit einer Person Widerstandsressourcen zur Verfügung stehen, um mit Belastungssituationen umzugehen. Beschäftigte mit einem hohen Maß an Handhabbarkeit können unerwartete Ereignisse und Schicksalsschläge bewältigen, ohne in eine Opferrolle zu verfallen [37]. Die Sinnhaftigkeit widerspiegelt das Ausmaß, in dem eine Person das eigene Leben als sinnvoll erachtet und der Ansicht ist, dass es sich lohnt, sich gestellten Herausforderungen und Aufgaben entgegenzustellen und diese zu bewältigen [37].

Eine Studie mit über 4000 deutschen Beschäftigten im Gesundheitswesen konnte aufzeigen, dass ein höherer SoC mit weniger Angst- und Depressionssymptomen einhergeht [38]. Auch bei Anästhesisten auf einer Intensivstation war SoC ein wichtiges Korrelat für die allgemeinen psychischen Gesundheitsprobleme sowie für die Ausprägung von Symptomen einer posttraumatischen Belastungsstörung [39].

**Figure 7:**
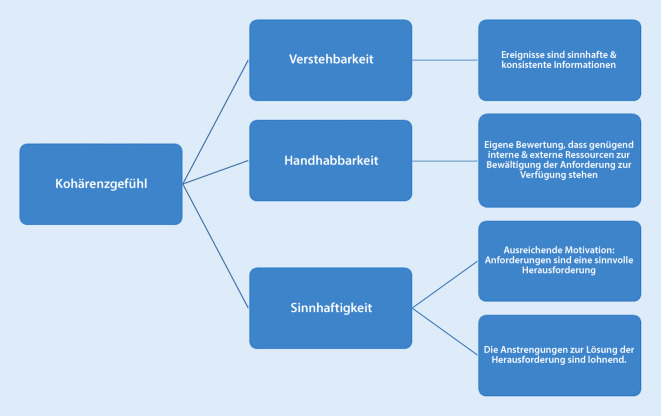

Studien belegen, dass Veränderungen der Arbeitsumgebung sich auf die Ausprägung des Kohärenzgefühls auswirkten [40]. Arbeitsbezogenes Kohärenzgefühl (Work-SoC) wird als die wahrgenommene Verstehbarkeit, Handhabbarkeit und Sinnhaftigkeit der aktuellen Arbeitssituation einer Person verstanden, die durch die Interaktion individueller Charakteristika (Persönlichkeit und individuelle Ressourcen) sowie Charakteristika der Arbeitsumgebung (Strukturen und Prozesse) beeinflusst wird (Abb. 7; [41]).

Hohes Kohärenzgefühl zeigte auch positive Einflüsse auf arbeitsbezogene Ressourcen [42–44] und Wahrnehmung eines guten Arbeitsklimas [42] bzw. einer besseren *sozialen Unterstützung* durch Kollegen [45]. Personen mit einem stark ausgeprägten Kohärenzsinn beurteilen äußere Belastungsfaktoren weniger als belastend und sehen diese als positive stimulierende Herausforderung. Personen mit niedrigem Kohärenzsinn sehen diese eher als belastenden Stressor. So wurde die rasante Entwicklung digitaler Besprechungen in verschiedenen Konferenzformaten (z. B. Webex, Zoom, Microsoft Meetings) und der onlinebasierten Lehre in der Chirurgie von einigen als positive stimulierende Herausforderung und persönliche Weiterentwicklung wahrgenommen, von anderen als ziemlich belastende (eher fast lähmende) Situation.

- Nach besonders gravierenden Ereignissen (z. B. in der Chirurgie Tod eines Patienten) und je nach Bewältigungsmöglichkeiten ist das *Erleben sozialer Unterstützung* ein entscheidender Faktor für die Verarbeitung der Belastungssituation [46]. Gemeint ist hier nicht nur die private, sondern auch die soziale Unterstützung im beruflichen Umfeld. Auch in der bereits oben erwähnten Studie mit deutschen Beschäftigten im Gesundheitswesen konnte belegt werden, dass eine höhere soziale Unterstützung mit weniger psychischen Symptomen einherging [38]. Generell gilt die Notwendigkeit, Belastungen im beruflichen Alltag auch im Arbeitsumfeld aufzuarbeiten [47].
- Eine *hohe Selbstwirksamkeitserwartung* wird während der eigenen Lerngeschichte entwickelt, indem positive Erfolgserfahrungen durch die eigene Kompetenz, positive Bewertungen Dritter und Beobachtungslernen stattfinden [48]. Dieses Konzept ist an die sozial-kognitive Theorie von Bandura angelehnt [48, 49]. Personen mit hoher Selbstwirksamkeitserwartung empfinden Stressoren eher als Herausforderungen, nutzen aktive und problemorientiere Bewältigungsstrategien und weisen ein höheres Durchhaltevermögen bei Hindernissen auf [31]. Reviews konnten einen positiven Zusammenhang zwischen hoher Selbstwirksamkeitserwartung und besserer psychischer Anpassung nach kritischen Ereignissen beobachten [50, 51], so auch bei niederländischen Militärangehörigen [52] oder bei australischen Feuerwehrmännern [53].

### Resilienzförderung – Real-life-Beispiele für die Umsetzung im beruflichen Alltag in der Chirurgie

Berufliche Resilienzförderung bedeutet der individuelle Umgang mit Herausforderungen, beruflichen Stressoren unter Berücksichtigung des Umfeldes (z. B. bestehen gerade Schicksalsschläge im privaten Kontext). Ziel ist es, die Gesundheit zu erhalten, Lebensqualität und -freude, Kreativität und Motivation zu steigern [24].

Um Resilienz zu stärken, bedarf es der Identifizierung bestehender Probleme und die Herleitung von Fragestellungen [24], bspw.:

- Sie fühlen sich durch Ihre Arbeit gestresst, ausgebrannt, überfordert?
- Ein Ereignis auf der Arbeit, z. B. komplikationsreicher Verlauf einer Behandlung, Tod eines Patienten, führte zu einer Krise?
- Sie fühlen sich fremdbestimmt?
- Sie haben Ängste und Sorgen?
- Sie möchten Ihr Leben so gestalten, dass es Ihnen und Ihren Angehörigen wieder besser geht?
- Sie fühlen sich, als ob Sie eine To-do-Liste abarbeiten?
- Sie möchten gelassener und dickhäutiger werden? Oder sich besser abgrenzen können?
- Sie möchten Ihre Bedürfnisse besser erkennen?
- Sie möchten selbstfürsorglicher sein?
- Sie wollen herausfinden, was im Leben wirklich zählt?
- u. a.

Nachfolgend werden sechs Resilienzfaktoren aufgeführt, die evidenzbasiert multimodal im Resilienz-Coaching angewandt werden [54]. Je nach identifizierter Kernfrage, eigener Veranlagung und Kompetenzen bedarf es individuelle oder zielgruppenspezifische Angebote.

Die Selbstregulationsfähigkeit ist übergeordnet und beinhaltet innerpsychische Prozesse und bietet Einflussmöglichkeiten und bedarf Impulskontrolle, Frustrationstoleranz und Affektregulation [54]. Es ist eine Fähigkeit, bewusst und willentlich auf uns Einfluss zunehmen und mit ausreichendem Wissen, welche Techniken notwendig sind. Das Ziel sollte gesundheitsförderlich sein und das Wohlbefinden steigern. Typische Ansätze sind die Zulassung positiver Emotionen und die Akzeptanz negativer Situationen:

- Akzeptanz negativer Situationen
  - Frustrane Reanimation in der Notaufnahme kann schicksalshaft sein → Sichtung der Akte und Debriefing im Team notwendig, um zu erkennen, dass alles richtig gemacht wurde und der Verlauf schicksalshaft bleibt.
  - Meine Entscheidung im Dienst führte zu einem Fehler und einem komplikationsreichen Verlauf → „human factor“ als Waffe gegen „human error“ → „crisis resource management“ (CRM) und Teamarbeit als Sicherheitskultur akzeptieren und daran teilnehmen; Akezptanz von Checklisten und SOP [55].
  - Dienst ist Dienst, auch wenn die schwierige Flexülenanlage gerade in meinem Dienst passiert.
- Akzeptanz negativer Emotionen
  - Natürlich kann mich der komplikationslose Verlauf oder der Tod des Patienten berühren – ich bin in erster Linie ein Mensch und Empathie ist eine unverzichtbare Kernkompetenz des Arztberufes.

Optimismus ist bekannt geworden durch die positive Psychologie nach Seligman [56], d. h. wir können das Glas halb voll oder halb leer sehen. Ein optimistischer Blick kann erlernt werden und hilft uns dabei, soziale Kontakte zu binden und zu pflegen [54].

Typische Beispiele sind:

- Das Patientenaufkommen nimmt kein Ende. → Der Dienst wird heute schnell vorbeigehen.
- Fehlende Bettenkapazitäten führen dazu, ggf. Patienten vorzeitig zu entlassen. → Auch gut, dann müssen keine Operationen verschoben werden und ich kann meine Fertigkeiten trainieren. Zuvor fordere ich aber ein Briefing mit meiner Führungskraft.

Die Stärkung des sozialen Netzes und der Beziehungen gehört ebenfalls zu den wichtigsten Resilienzfaktoren. Im chirurgischen Setting ist es der kollegiale Austausch zwischen verschiedenen hierarchischen Ebenen.

- Führungskräfte sollten eine „no blame no shame culture“ implementieren. Uns sollte bewusst werden, dass niemand gerne Fehler macht, insbesondere wenn die Gesundheit von Patienten involviert ist. Grobfahrlässige Handlungen stehen hier nicht zur Diskussion.
- Besondere Fallstricke bedürfen das bereits oben erwähnte CRM. Dabei sollte jede Art von Fehlern, Irrtum oder potenzieller Irrtum (sofort) erkannt werden und ohne Angst vor Bestrafung evaluiert werden.
- Aus der Komfortzone herauskommen und Probleme und offene Fragen offen diskutieren → Fallbesprechungen mit allen hierarchischen Ebenen sind hier ein guter Ansatz.
- Gerechtfertigte Kritik an der Behandlung des Patienten zulassen, reflektieren und davon lernen.
- Die Pflegekraft überschreitet ihre Kompetenzen und möchte mir vorschreiben, wie ich die (Notfall‑)Behandlung durchführe. → Als Arzt eine gerechtfertigte Speak-up-Kultur zulassen, denn eine situationsgerechte Wahrnehmung kann schwanken. Speak-up bedeutet hier, dass Unterstellte sagen, was sie wirklich denken und somit zur Sicherheitskultur beitragen [57]. Speak-up-Kultur sollte dann gelten, wenn Schweigen gefährlich werden kann, z. B. durch den Tod eines Patienten.

Selbstwirksamkeit ist ein weiterer Resilienzfaktor. Selbstwirksamkeitserwartungen werden durch Lernerfahrungen, erhaltenes Feedback und durch die Beobachtung Dritter gebildet [55]. Die Selbstwirksamkeitserwartung ist umso größer, umso mehr Vertrauen in die eigenen Kompetenzen steckt. Hierbei geht es auch um das Lösen herausfordernder Situationen, die neu für uns sind und damit auch Anstrengung und Ausdauer erfordern.

- Jede neue Situation bedarf es zu evaluieren, Kompetenzen zu kennen und ggf. rechtzeitig Hilfe anzufordern. Das gilt insbesondere für Tätigkeiten in der Notaufnahme. → Anforderung des Hintergrunddienstes bei Rückfragen oder fehlenden Kompetenzen ist keine Schande. Multitasking während einer Notbehandlung ist schwierig und kann zu Fehlern führen. Die Behandlungsqualität in Notaufnahmen schreibt sowieso einen Facharztstandard vor [58]. Dies sollte auch vom Hintergrunddienst akzeptiert werden. Auch hier gilt Dienst ist Dienst.

Ein zukunfts- und lösungsorientierter Ansatz als weiterer Resilienzfaktor bietet Zugang zu möglichen Lösungen und somit Möglichkeiten, künftig in Krisen oder stressigen Herausforderungen selbstwirksam und handlungsfähig zu bleiben [54]. Studien belegen, dass die Akzeptanz von Negativereignissen zur signifikant besseren Lebensqualität, Wohlbefinden und besserer psychischer Gesundheit führt [59]. Grundlegend ist die eigene Erkenntnis, was wir selber beeinflussen können oder eben nicht. Im Vordergrund steht hier auch die Nutzung vorhandener Ressourcen.

- Der angekündigte Notfallpatient bereitet mir jetzt schon Sorgen. → In erster Linie Führungsposition annehmen und akzeptieren, jedoch dabei wissen, dass Kommunikation und Teamarbeit ein unverzichtbarer Teil der Behandlung, des Outcomes des Patienten und des allgemeinen Wohlbefindens danach ist. Reden Sie mit Ihrem Team. Es gilt, dass Fachwissen und Fertigkeiten aller Teammitglieder zu erkennen und zu nutzen.

Ein weiterer Resilienzfaktor, Sinn- und Werteorientierung, ist das Fundament. Es kommt häufig in „ruhigeren“ Settings, z. B. auf Station zum Tragen. Eigene Werte und Bedürfnisse sowie die Sinnvorstellungen sind Motivator für unser Handeln und Entscheidungen. Was wir wertvoll erachten, entscheidet wie wir uns im Alltag verhalten.

Ergebnisse eines systematischen Reviews, welches 29 Studien von Pflegekräften und Ärzten inkludierte, deutet darauf hin, dass Coaching von Achtsamkeit und kognitive Verhaltenstherapie Stress, Ängste und Depressionen wirksam verringerten. Kurzinterventionen, wie bspw. tiefes Atmen und Dankbarkeit, vorteilhaft sein könnte [60]. Eine Studie untersuchte Resilienztraining, durch psychosoziale Fachkräfte in Krankenhäuser als Unterstützung wichtige Herausforderungen (z. B. SARS-CoV-2-Pandemie) zu meistern und Burnout entgegen zu wirken [61]. Teilnehmer einer weiteren Studie berichteten, dass Resilienz-Coaching den Krankenhausmitarbeitern die Möglichkeit bot, Kontakte zu knüpfen, sie zu ermutigen, sich um ihr persönliches Wohlbefinden zu kümmern, und ihnen praktische Fähigkeiten zur Bewältigung vermittelte [62].

Zusammenfassend ist erkennbar, dass Resilienz-Coaching ein Modell zur Unterstützung von Kollegen in Krankenhäusern ist. Es bedarf weitere Forschung, um festzustellen, wie bestimmte berufliche Settings am besten davon profitieren können.

### Resilienz stärken durch Stressmanagement und kohärente Führung

**Figure 8:**
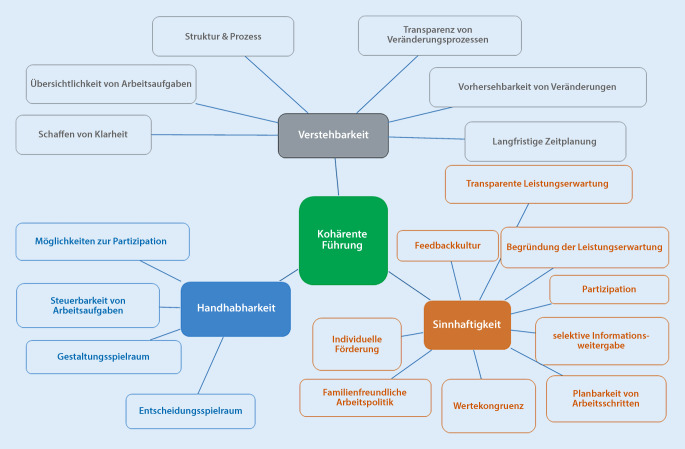

Interaktionsdynamik im Sinne einer kohärenten Führung gegenüber Mitarbeitenden kann einen großen Stellenwert einnehmen. Es ist jedoch keine fachliche Fähigkeit der Führungskraft, sondern bedarf einer hohen Kompetenz in der Interaktion mit Mitarbeitenden. Es schließt zwei oder mehrere Personen ein, ist zweckbestimmt, führt zur sozialen Einflussnahme und ist ein Kommunikationsprozess [63]. Mögliche Inhalte einer kompetenten Führung sind in Abb. 8 dargestellt.

**Figure 9:**
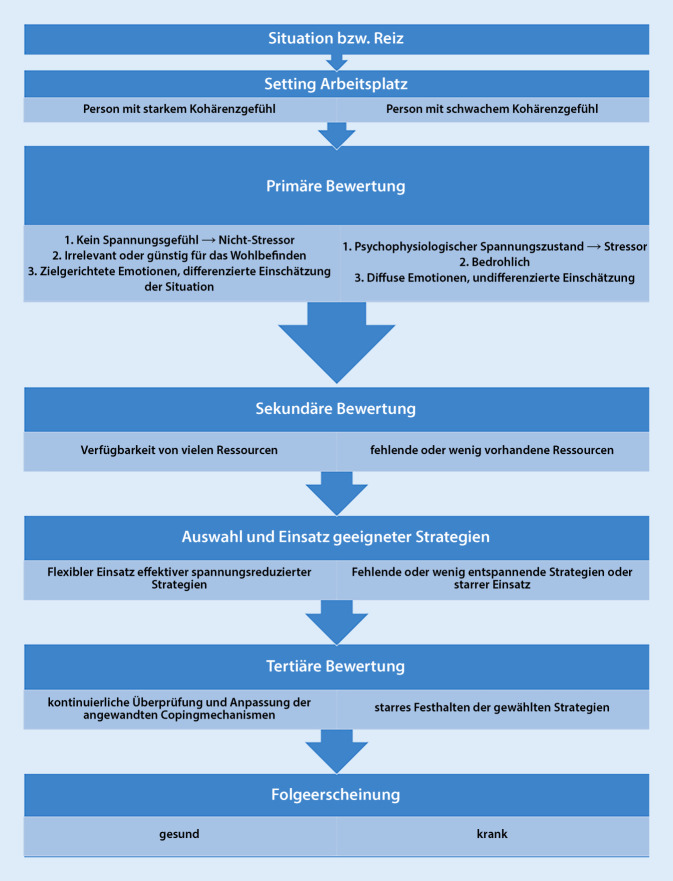

Das Kohärenzgefühl kann nicht als Coping-Strategie betrachtet werden, sondern als Voraussetzung von Personen, die diese befähigt, flexibel auf Anforderungen zu reagieren [37]. Schematisch ist dies in Abb. 9 dargestellt. Personen mit einem stark ausgeprägten Kohärenzgefühl sind eher in der Lage, verschiedene Strategien der Stressbewältigung für sich zu mobilisieren und sich im Gesundheits-Krankheits-Kontinuum verstärkt in Richtung Gesundheit zu bewegen [37]. Die Gesundheit und das Wohlbefinden von Mitarbeitern hängen somit maßgeblich von der Führungsqualität ab [64].

Zu Beginn einer Bewertung von Stressoren sollte jedem bewusst sein, welche Stressreaktion der Betroffene aufweist und in welcher Anspannungsphase sich dieser befindet. Dabei stellt sich z. B. bei Überlastung von Mitarbeitenden die Frage, ob zunächst Pausen eingehalten werden und ob es Phasen des Innehaltens oder unter Rufbereitschaft sind. Das Wissen, dass Krisen in der Regel zeitlich limitiert sind, wirkt dem Stressempfinden positiv entgegen.

Akzeptanz für Veränderungen ist eine wichtige Voraussetzung für das Resilienztraining. Resilienztraining zielt nicht darauf ab, den Zustand blind zu akzeptieren, also eine Anpassung des Menschen an die Gegebenheit zu erzielen [65]. Dabei orientiert sich das Training an den Bedürfnissen der Teilnehmenden oder des Unternehmens (sog. organisationale Resilienz) und kann nicht pauschalisiert werden. Unterstützend kann es ein Wohlfühlort sein. Dabei empfiehlt es sich, auf jeweils einzelne Punkte zu fokussieren. Typische Elemente der organisationalen Resilienz sind:

1. geteilte Vision und klare gemeinsame Zielformulierung auf jeder Hierarchieebene,
2. Verstehen und Beeinflussung des internen und externen Umfeldes, was Möglichkeiten zur Einflussnahme schafft,
3. effektive und ermutigende Führungskultur,
4. Schaffung einer resilienzfördernden Kultur mit gemeinsamen Überzeugungen und Werten, positiven Einstellungen und kommunikationsbezogenen Verhaltensweisen wie Begründung der Leistungserwartung, selektiver Informationsweitergabe und direkter Kommunikation,
5. Lernkultur implementieren durch Teilen von Information und Wissen,
6. Entwicklung und Verfügbarmachen von Ressourcen durch Schulungen, Hilfsmittel,
7. Koordination von Unternehmensbereichen mit Entwicklung strategischer Ziele (z. B. Bildung von Abteilungen wie Human Resources, Betriebliche Gesundheitsförderung usw.),
8. kein Stillstand, sondern kontinuierliche Verbesserung zulassen, z. B. durch Evaluierung von Ergebnissen und Erfahrungen im Sinne eines lernenden Unternehmens,
9. Antizipierung und frühzeitiges (aktives) Management von Veränderungen.

## Stärken und Limitationen des narrativen Reviews

Im Rahmen dieses Reviews werden Literatur und eigene Erfahrungen kombiniert und typische Belastungsfaktoren in der Chirurgie dargestellt. Es werden Empfehlungen für die Chirurgie hinsichtlich resilienter Arbeitsweisen formuliert. Limitierend zu sehen ist, dass im Rahmen des narrativen Charakters nicht systematisch nationale und internationale Literatur berücksichtigt werden konnte.

## Schlussfolgerungen

Die Konsolidierung der Resilienz als beachtenswerter Aspekt der Mitarbeiterführung und im interkollegialen Umgang sollte sich auf arbeitsplatzbezogene Ansätze stützen zur Stärkung der Bewältigungsmechanismen gegenüber Arbeitsbelastungen. Arbeitsplatzbedingte Belastungen sollten auch – durchaus auch als elementare Leitungsaufgabe – unternehmensintern wahrgenommen, angesprochen und ihren entgegengewirkt werden.

Insgesamt sind Daten über die Resilienz der Chirurgen bzw. über Interventionsstudien in der Resilienzforschung im Setting Chirurgie limitiert und bieten eine weitere Forschungslücke. Resilienztraining – auch klar angezeigt im „robusten“ medizinisch-operativen Fach Chirurgie – ist immer individuell und sollte nicht pauschalisiert werden.

Die Autoren kommen zum Fazit, dass in der Chirurgie eine gezielte Resilienzstärkung möglich und notwendig ist. Die moderne Resilienzforschung und der vorliegende Beitrag adressiert das so wichtige Thema. Es erscheint auch sinnvoll, da der chirurgische Nachwuchs ausbleibt. Resilienzstärkung beginnt jedoch schon während des Humanmedizinstudiums und sollte im Rahmen der Assistenzarztausbildung fortgeführt werden. Nicht zu vergessen ist die kontinuierliche Resilienzstärkung während des Facharztstatus oder darüber hinaus. Eine grundlegende Basis dafür stellen nachfolgende Eckpunkte dar:

- eine vice versa interkollegiale und interprofessionelle Unterstützung,
- eine verantwortungsvolle Vorgesetztenrolle,
- eine kompetente und wertschätzende Mitarbeiterführung auf allen Ebenen und
- eine notwendige und sinnvolle psychologische Begleitbetreuung.

Es bedarf weiterer Forschungsarbeiten auf diesem Gebiet, um zu erfahren, welche Methoden für die Chirurgie am besten geeignet sind.

Betriebsärztliche Strukturen sollten als rechtliche Rahmenbedingung implementiert und je nach Bedarf stärker ausgebaut werden. Mitarbeitende sollten auf das Angebot betriebsärztlicher Maßnahmen und Prävention sensibilisiert werden. Das Thema sollte nicht negiert oder tabuisiert werden, sondern als Teil einer Präventionskultur eingeführt und gestärkt werden. Das gilt vor allem auch für notwendige und sinnvolle psychologische Interventionen zur Stärkung der Resilienz.

## Footnotes

1 Die in dieser Publikation verwendeten Personenbezeichnungen beziehen sich immer gleichermaßen auf alle Geschlechter. Auf eine Doppelnennung und gegenderte Bezeichnungen wird zugunsten einer besseren Lesbarkeit verzichtet.
